# Corrosion Screening of Greenhouse Structural Steel Pipes Using Cold Infrared Thermography

**DOI:** 10.3390/s26185719

**Published:** 2026-09-09

**Authors:** Junghwa Park, Jaehun Lee, Gunhui Park, Eunji Jung, Sungwook Yun, Jaesung Park

**Affiliations:** 1 Department of Bio-Industrial Machinery Engineering, College of Natural Resources and Life Science, Pusan National University, Miryang 50463, Republic of Korea; junghwa@pusan.ac.kr (J.P.); wogns011025@pusan.ac.kr (J.L.); gunhui@pusan.ac.kr (G.P.); quberry9548@pusan.ac.kr (E.J.); 2 Smart Farm Development Division, National Institute of Agricultural Sciences, Rural Development Administration, Jeonju 54875, Republic of Korea; wookswooks@korea.kr; 3 Major in Natural Resources Systems Engineering, Pusan National University, Yangsan 50612, Republic of Korea; 4 Life and Industry Convergence Research Institute, Pusan National University, Miryang 50463, Republic of Korea

**Keywords:** corrosion-group discrimination, direct and indirect cooling regions, cooling and recovery phases, linearity indices, time constant

## Abstract

Greenhouse structural steel pipes are susceptible to internal and external corrosion under hot and humid service conditions, leading to cross-sectional loss and reduced structural strength. This study proposes a cold infrared thermography method for screening corroded pipes and identifies effective indicators for different cooling-affected regions. Experiments were performed using three non-corroded and five salt-spray-corroded SPVHS-grade specimens and three field-corroded specimens. After a cooling stimulus was applied using an ice pack, time-series mean surface-temperature data were acquired using an infrared camera. For the direct cooling region, the recovery phase was fitted with an exponential model to estimate the time constant (*τ*). For the indirect cooling region, located approximately 50 mm from the direct cooling region, the cooling phase was fitted with a linear model to obtain *R*^2^ and the root mean square error (RMSE). Mean *τ* was consistently higher in the non-corroded group than in the combined corroded group across all four environmental conditions. The linearity indices in the indirect cooling region showed only exploratory, condition-dependent group differences. These findings suggest that the recovery time constant may support corrosion screening under controlled conditions.

## 1. Introduction

Greenhouse structural steel pipes are essential framing members in lightweight agricultural structures and are susceptible to corrosion caused by prolonged exposure to high temperature and humidity, condensate, fertilizer and pesticide constituents, and airborne salts in coastal areas [1,2,3]. Even pipes that appear sound may contain concealed defects, including wall thinning, oxide-layer formation, and localized corrosion at joints [4,5]. Such corrosion reduces the pipe cross-section and mechanical strength [6], thereby increasing the risk of structural collapse under heavy snow or strong winds. Early diagnosis and timely replacement are therefore required. Major non-destructive testing methods applicable to steel-pipe corrosion include visual inspection, hammer sounding, ultrasonic thickness measurement, and eddy-current testing [7]. Visual inspection and hammer sounding depend strongly on inspector experience and are difficult to implement quantitatively and reproducibly. They are also limited in detecting early-stage corrosion or localized corrosion concealed by connecting components. Ultrasonic thickness measurement is effective for confirming actual wall loss; however, it is a contact-based, pointwise technique, and full-surface inspection requires an impractically large number of measurements. Its results are also sensitive to maintaining adequate coupling through the couplant. Eddy-current testing and magnetic flux leakage testing have limited field applicability because of lift-off sensitivity, namely signal distortion and instability caused by variations in the sensor-to-surface distance, and operational complexity [8,9]. The common limitations of conventional techniques can therefore be summarized as difficulty in assessing large areas using point- or line-based measurements, reduced workability due to contact or close-proximity requirements, and challenges in ensuring measurement reproducibility. A quantitative, non-contact, area-based non-destructive testing method is consequently needed.

Infrared thermography (IRT) is a non-contact technique that images thermal radiation emitted from an object’s surface and is generally classified as passive or active IRT [10]. Passive IRT observes naturally emitted radiation without applying an external thermal stimulus, whereas active IRT analyzes surface-temperature changes during transient heat transfer after external thermal excitation. Because subsurface defects can be inferred indirectly from their thermal-response patterns, active IRT has been widely applied to detect delamination, voids, corrosion, and other damage in metals and composite materials [11,12,13,14]. Thermography provides area-based, non-contact measurements and can acquire high-resolution time-series temperature data in real time, offering substantially greater information density than contact-based pointwise non-destructive testing. Nevertheless, thermography does not directly measure material thickness and is affected by emissivity, ambient convection, and reflected radiation. Standardized measurement conditions and integration with precision inspection techniques are therefore required.

Most active-thermography studies have used heating excitation from halogen lamps, flash lamps, or induction heating. These approaches are readily controlled because they transfer thermal energy efficiently and are supported by integrated equipment configurations. Cooling-based excitation has been investigated far less frequently; however, its principal advantages are operational safety and the minimization of thermal damage. In environments containing abundant combustible materials, such as plastic-film greenhouses, high-temperature excitation sources introduce a fire hazard. Cooling excitation poses no fire risk and does not thermally damage the material, making it well suited to field application in greenhouse environments. It is also practical because simple and widely available cooling media, such as cooling sprays, can be used with relatively low operational complexity.

Doshvarpassand et al. [15] employed cooling excitation to detect corrosion in metallic structures in environments such as liquefied natural gas plants, where flammable or explosive gases may be released. Using a cooling spray as the excitation source, they developed an integrated pipeline for detecting internal metal-loss defects and quantitatively estimating defect depth. Their initial work proposed a mobile scanning prototype that combined a cooling spray with an infrared camera and introduced dynamic reference reconstruction (DRR). The method successfully detected defects located as deep as 5 mm below the measured surface, with a minimum detectable metal loss of 37.5%. In a subsequent study, Doshvarpassand and Wang [16] extended the method from defect detection to quantitative characterization by numerically estimating defect depth and diameter. They proposed an analytical model that accounted for three-dimensional lateral thermal diffusion away from defect edges and compared the pattern similarity between experimental control time-series data and analytical-model data using dynamic time warping (DTW). The method successfully approximated metal-loss depths of up to 50%. Collectively, these studies indicate that, when combined with advanced data-processing methods, cold infrared thermography can provide detection performance comparable to heating-based active thermography while offering superior safety and field applicability.

Cold infrared thermography may therefore provide a safe alternative for detecting corrosion in greenhouse structural steel pipes. However, measurement procedures and thermal-response indicators for cooling excitation have not yet been standardized or widely adopted. This study aimed to design a cold infrared thermography method for corrosion screening of structural steel pipes and to identify the principal thermal-response indicators for discrimination and applicable operating conditions for different cooling-affected regions. Accordingly, this study provides a proof-of-concept evaluation of whether cooling-induced thermal-response indicators can distinguish non-corroded and corroded specimens under controlled experimental conditions. The temperature-time responses were analyzed separately in a direct cooling region, where the cooling stimulus was applied, and an indirect cooling region, where the effect of cooling was transferred through the pipe. The time constant *τ*, representing the recovery rate during the temperature recovery phase, was evaluated in the direct cooling region. In the indirect cooling region, the coefficient of determination *R*^2^ and root mean square error (RMSE) were introduced as linearity indices describing the shape of the cooling curve. These indicators were then used to assess the feasibility of distinguishing non-corroded and corroded specimens.

## 2. Materials and Methods

### 2.1. Specimens

The specimens were SPVHS-grade galvanized steel pipes for plastic-film greenhouses, corresponding to the KS D 3760 [17] specification, with an outside diameter of 25.4 mm and a wall thickness of 1.5 mm. A total of 11 specimens were classified into three groups according to corrosion condition: non-corroded (NC), salt-spray-corroded (SC), and field-corroded (FC). The specimen classifications and characteristics are summarized in Table 1. The NC group comprised three specimens prepared by cutting newly purchased 1000 mm steel pipes into 500 mm sections. The SC group comprised five pipes of the same specification as the NC specimens that were subjected to a salt-spray test in accordance with KS D 9502 [18]. All five specimens were placed in the test chamber simultaneously, and one specimen was removed every 24 h to produce stepwise exposure durations (SC1: 24 h, SC2: 48 h, SC3: 72 h, SC4: 96 h, and SC5: 120 h). Zinc corrosion products and white rust had formed over the entire surface of every SC specimen. The FC group comprised three discarded pipes that had been used in actual greenhouses for more than 10 years and exhibited mild red rust. Whether these pipes met the SPVHS specification was unknown, and their lengths ranged from approximately 570 to 710 mm. Soil and dust adhering to the field-corroded specimens were wiped from the surface before testing. The salt-spray exposure duration was treated as an exposure condition and not as a quantitative measure of corrosion severity. Similarly, the actual corrosion severity of the FC specimens was not quantified. Therefore, the NC, SC, and FC classifications represent specimen condition and exposure history rather than quantitatively defined levels of corrosion severity.

**Figure 1:**
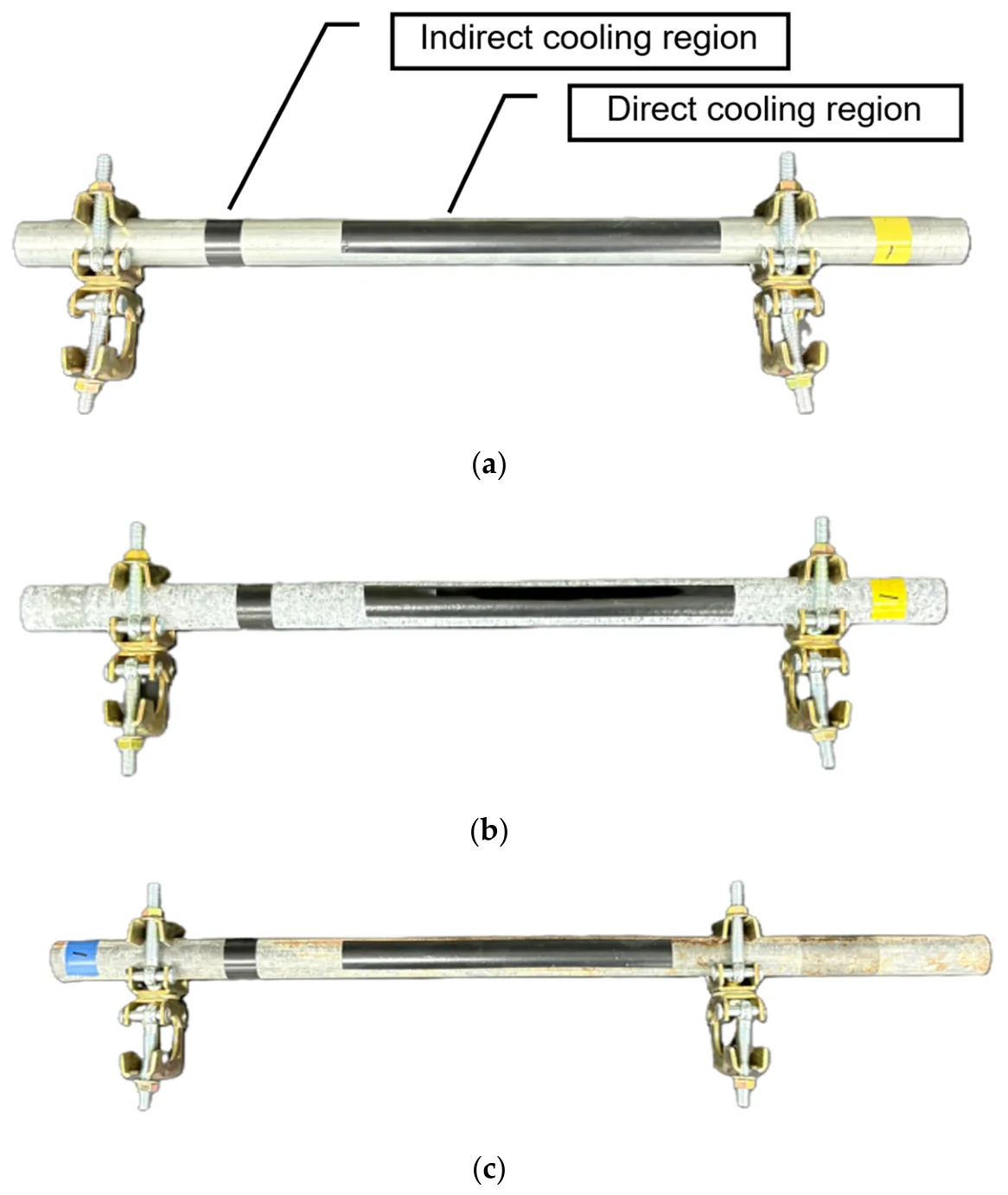
A specimen secured with a clamp, showing the direct and indirect cooling regions indicated by insulation tape: (**a**) Non-corroded specimen; (**b**) Salt-spray-corroded specimen; (**c**) Field-corroded specimen.

Two clamps were attached to each specimen at positions that did not overlap the regions of interest (ROIs). The clamps prevented specimen rotation and displacement and eliminated direct contact with the background surface, thereby minimizing background heat transfer. They also served as handles during specimen transport and reduced temperature disturbance caused by human contact. The prepared specimens are shown in Figure 1.

### 2.2. Experimental Equipment and Environment

#### 2.2.1. Infrared Thermal Imaging System

Infrared thermal imaging was performed using a FLIR T560 camera (Teledyne FLIR, Wilsonville, OR, USA). Its principal specifications are listed in Table 2. The recording frame rate was set to 3.75 fps, and the data were stored as radiometric Compressed Sequence (CSQ) files. FLIR Thermal Studio (version 2.0.73; Teledyne FLIR, Wilsonville, OR, USA) was used for data extraction.

**Figure 2:**
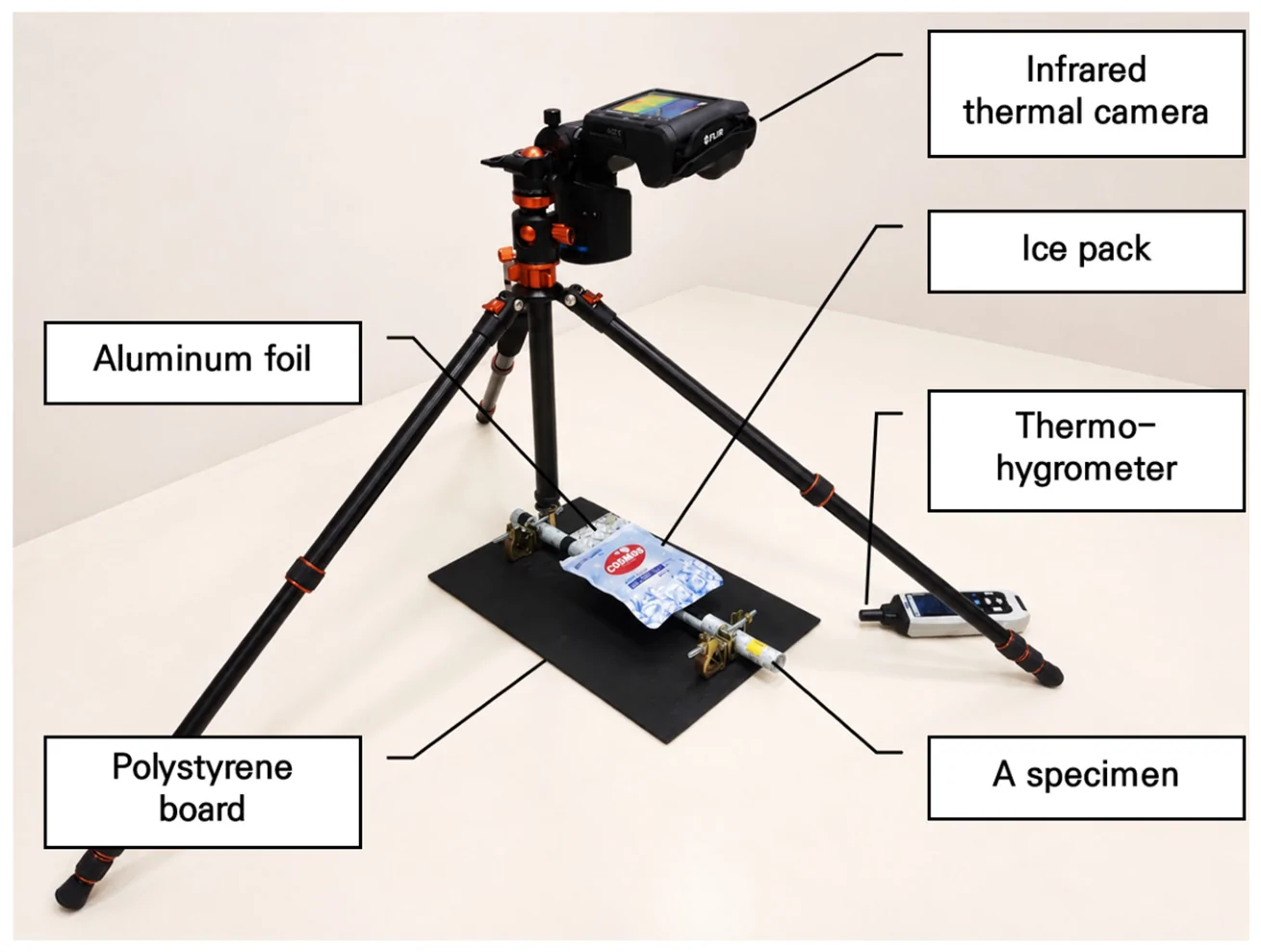
Infrared thermal imaging of the cooling and recovery process using an ice pack with the specimen positioned on the laboratory floor.

Thermal imaging was performed on the laboratory floor without separate control of heating, cooling, or ventilation during measurement. A black polystyrene insulation board was placed beneath the specimen to minimize thermal and reflected-radiation effects from the floor, and the specimen’s thermal response was then recorded with the infrared camera. The camera was mounted on a tripod and oriented vertically downward toward the specimen. The imaging distance was fixed at 0.5 m using the camera’s built-in laser distance meter. The experimental setup is shown in Figure 2.

#### 2.2.2. Thermographic Parameter Settings

Accurate thermographic temperature measurement requires correction for multiple radiation sources and the input of parameters such as target emissivity, reflected temperature, relative humidity, and atmospheric temperature. The surfaces of the non-corroded specimens were clean and glossy and therefore had very low emissivity, which could produce a large apparent temperature difference relative to corroded specimens even under identical conditions. To reduce uncertainty due to specimen emissivity, Scotch Super 88 electrical insulation tape (3M Company, St. Paul, MN, USA) was attached to every specimen ROI to establish a uniform emissivity condition. This procedure is recommended by Teledyne FLIR [19] as a method for increasing target emissivity. FLIR guidance specifies an emissivity of 0.96 for 3M Scotch Super 88; however, numerous thermography studies [20,21,22,23,24] have used a value of 0.95 for the same tape. Accordingly, emissivity was set to 0.95 in this study. The tape specifications are presented in Table 3.

Reflected-temperature correction was also performed according to Teledyne FLIR guidance [25]. Crumpled aluminum foil was placed near the specimen to produce diffuse reflection, and its apparent temperature was entered as the reflected-temperature value. The foil measured 100 × 90 mm, although only part of it was included in the thermal image. Its apparent temperature was obtained by setting the emissivity of the foil ROI (150 × 50 pixels) to 1.0 and reading the mean initial temperature at the center of the foil before cooling. Relative humidity and atmospheric temperature were measured with a digital thermo-hygrometer (model CHM-1; CAS, Yangju, Republic of Korea), and the initial values recorded before cooling were used as input parameters. The thermo-hygrometer had measurement ranges of −40–80 °C and 0–100% RH, accuracies of ±1 °C and ±3% RH, and resolutions of 0.01 °C and 0.01% RH, respectively.

#### 2.2.3. Cooling-Stimulus Source

A refrigerated gel ice pack measuring 150 × 190 mm with a nominal capacity of 300 mL was used as the cooling-stimulus source. This ice pack is designed to maintain a temperature near the phase transition temperature for an extended period, and condensation is effectively suppressed due to the non-woven fabric surface. Because the surface thermal response had to be observed reliably after cooling, excessive moisture deposition on the specimen was undesirable. Dry ice, cooling spray, and plastic-cased phase-change material (PCM) coolants were considered as alternative cooling media. Dry ice posed a risk of frostbite and was inconvenient to store; the discharge quantity and coverage of a cooling spray were difficult to control; and low-temperature PCM coolants operating near 0 °C were difficult to obtain. Ice packs and coolants with plastic surfaces are readily available, but condensation forms easily on such surfaces, causing moisture to adhere to the specimen and reducing experimental reproducibility. A nonwoven-surface ice pack was therefore selected to reduce condensation. The same ice-pack type and size were used throughout the experiments. The ice pack was reused only while it remained frozen and sufficiently rigid. The initial ice pack temperature was not measured instrumentally. Gloves were worn while handling the ice pack, and it was stored in an insulated bag immediately after each cooling stimulus. In some experiments, small amounts of moisture adhered to portions of the specimen surface because of the environmental conditions. The data were retained when no moisture-induced temperature difference was visible in the thermal images and the moisture dried naturally within 1–2 min during the recovery phase; otherwise, the experiment was repeated.

#### 2.2.4. Experimental Conditions

Experiments were conducted using a constant-temperature and humidity chamber (DOOSUNG, Gimhae, Republic of Korea) or under controlled room-temperature laboratory conditions. The principal specifications of the chamber are listed in Table 4.

The complete experiment was conducted under four environmental conditions. These conditions served as pretreatments for setting the initial specimen temperature and were used to examine how initial temperature affected cooling, temperature recovery, and corrosion discrimination. The chamber conditions were 27 °C/50% RH, 23 °C/50% RH, and 19 °C/50% RH, whereas the separate room-temperature condition ranged from 18 to 19 °C and 33–38% RH. Experiments were initially conducted under room-temperature control; chamber pretreatment was subsequently introduced to achieve stricter uniformity in initial specimen temperature. Under the chamber conditions, each experiment began within 1 min after the specimen was removed from the chamber. The 23 °C condition, based on the standard atmospheric temperature specified in ISO 554 [26], was used as the reference. Conditions 4 °C below and above this reference were then added: 19 °C, which approximated the room temperature at the time of testing, and 27 °C. These conditions are hereafter denoted as 27 °C, 23 °C, 19 °C, and RoomTemp 19 °C. The experimental conditions are summarized in Table 5. All specimens were preconditioned for at least 12 h under each environmental condition to reach thermal equilibrium.

### 2.3. Cold Infrared Thermography Measurement

#### 2.3.1. Definition of Regions of Interest (ROIs)

**Figure 3:**
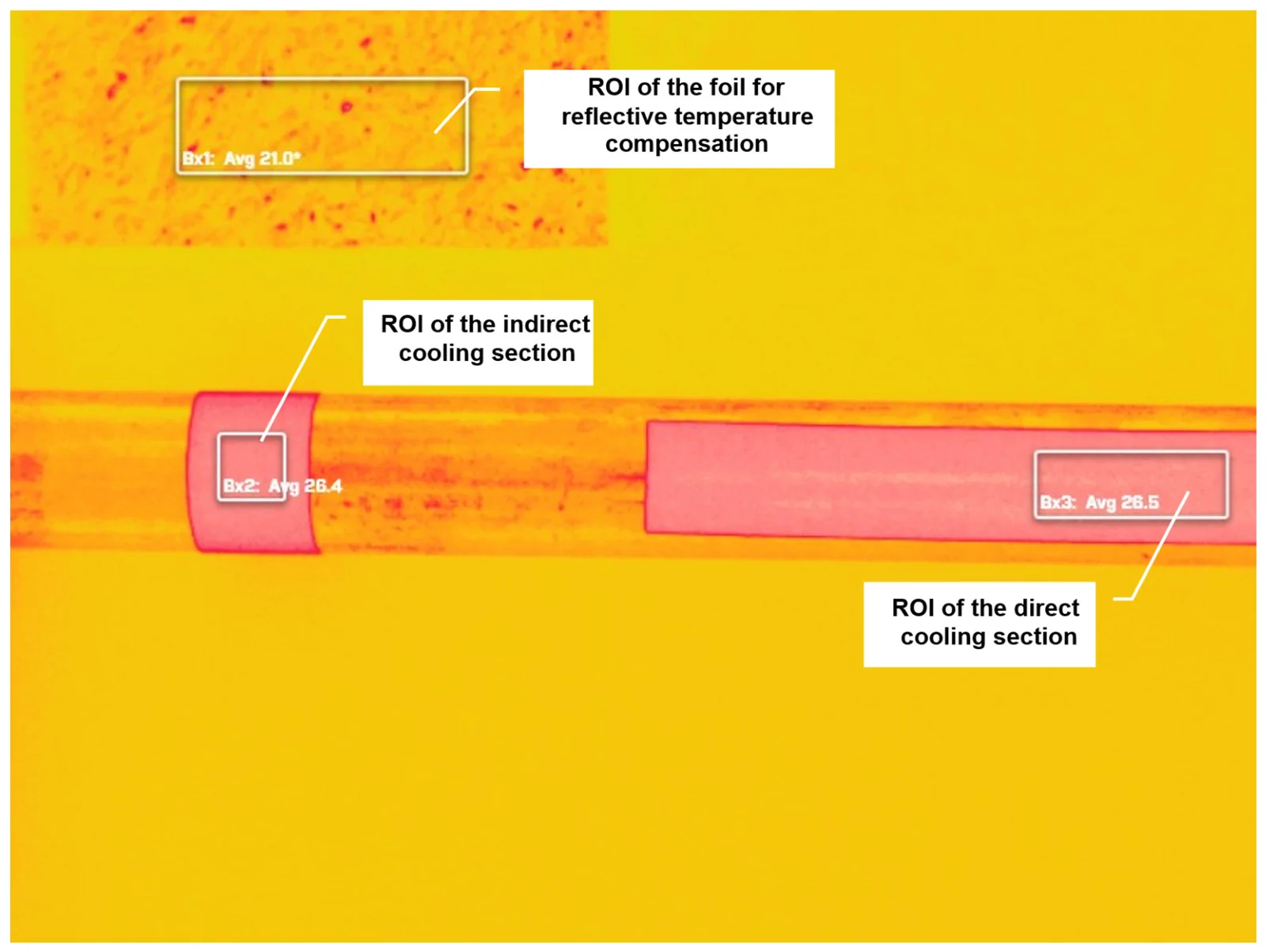
Configuration of regions of interest (ROIs) defined on the acquired infrared thermal data. The asterisk next to the mean ROI temperature indicates that local parameters differing from the default global settings were applied. For the aluminum foil ROI, the emissivity was individually set to 1.0.

The time-dependent surface-temperature response after cooling was analyzed separately in direct and indirect cooling regions. The direct cooling region was defined as the area in direct contact with the ice pack, whereas the indirect cooling region was defined as the adjacent area that was not in direct contact with the ice pack but was affected by heat transfer. As noted above, Scotch Super 88 insulation tape was applied to both regions on every specimen to equalize emissivity. In the direct cooling region, an approximately 200 mm strip of tape was attached along the pipe axis to indicate the ice-pack contact area. The indirect cooling region was marked by wrapping the tape once around the pipe circumference at a position approximately 50 mm from the direct cooling region; the resulting marked length was 19 mm, equal to the tape width. The ROI in the indirect cooling region was set to 35 × 35 pixels and positioned well within the circumferential tape area. The ROI in the direct cooling region was set to 100 × 35 pixels near the center of the ice-pack contact area. This rectangular ROI maintained the same height as the indirect ROI while reliably remaining within the analysis area despite slight specimen displacement or rotation. Both ROIs were included in the same frame, enabling the directly and indirectly cooled responses to be compared under identical experimental conditions. The ROI configuration is shown in Figure 3.

#### 2.3.2. Cooling-Stimulus Duration

**Figure 4:**
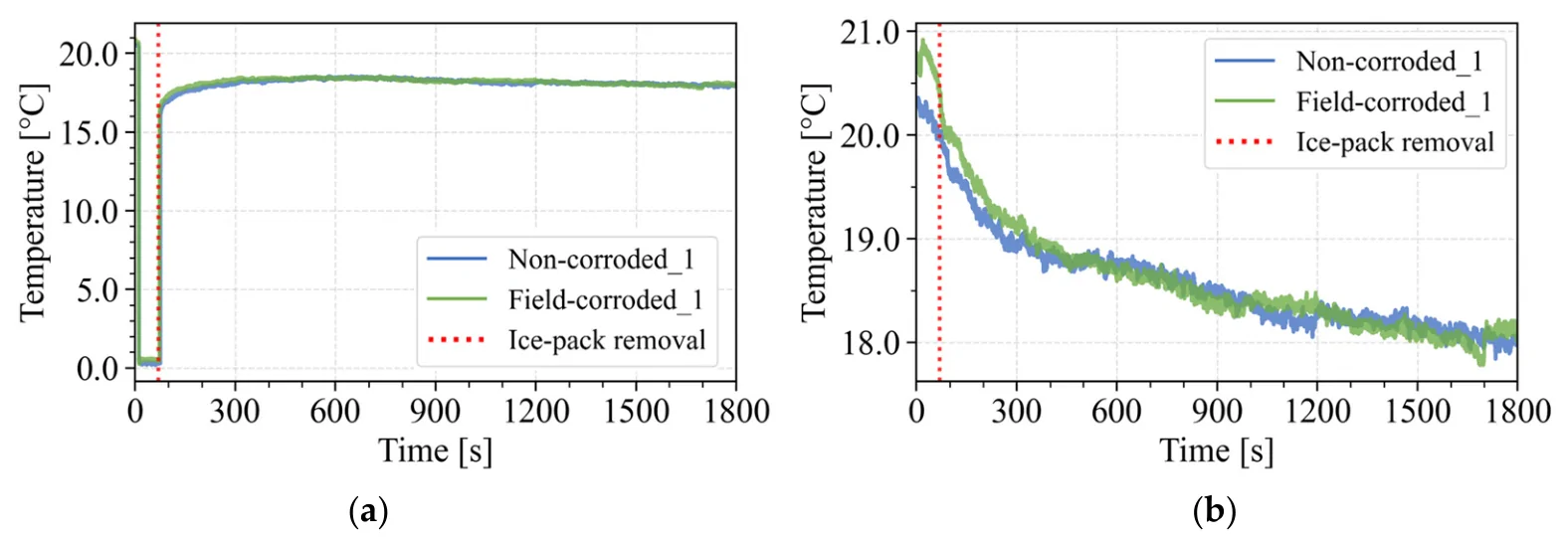

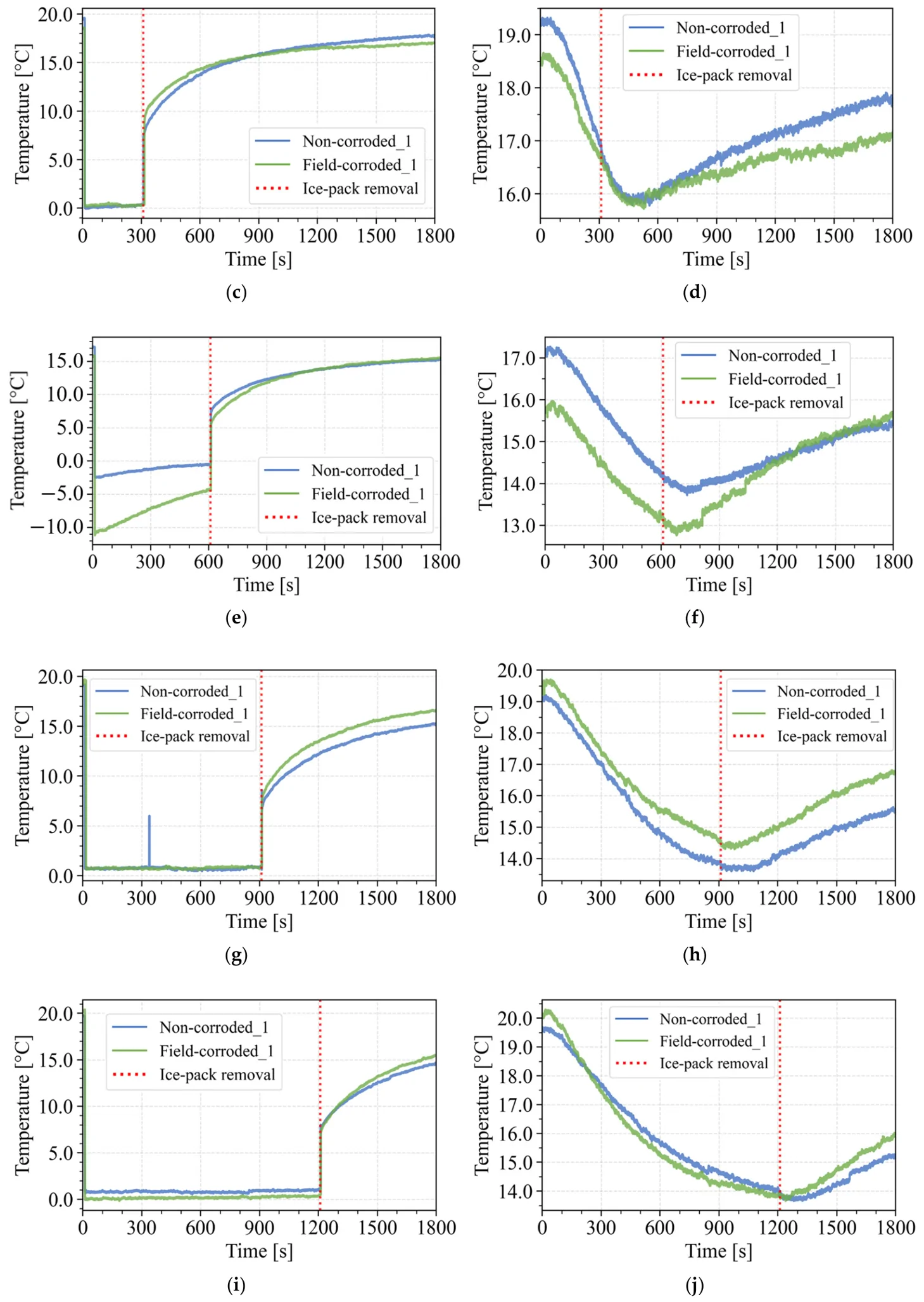
Mean surface-temperature trajectories of the direct and indirect cooling regions according to cooling duration for determining the optimal cooling time: (**a**,**b**) 1 min condition; (**c**,**d**) 5 min condition; (**e**,**f**) 10 min condition; (**g**,**h**) 15 min condition; (**i**,**j**) 20 min condition. For each pair, the (**left**) panel shows the direct cooling region and the (**right**) panel shows the indirect cooling region.

The total thermographic recording time was set to 30 min. This duration was selected to avoid reduced operational efficiency and changes in ambient conditions associated with excessively long acquisition, because measurements exceeding 30 min would have limited practical applicability when numerous locations must be inspected in a greenhouse. Preliminary tests evaluated ice-pack contact durations of 1, 5, 10, 15, and 20 min. The tests were conducted using non-corroded specimen 1 (NC1) and field-corroded specimen 1 (FC1) under laboratory conditions of 17–20 °C and 16–21% RH. Time-series mean surface temperatures in the direct and indirect cooling regions for each duration are shown in Figure 4. Because the ice pack obscured the direct cooling region during cooling, the stimulus duration was selected primarily on the basis of whether stable cooling and recovery phases could be obtained in the indirect cooling region.

With a 1 min cooling stimulus, the temperature in the indirect cooling region continued to decrease after ice-pack removal, and the recovery process could not be observed within the 30 min recording period. This behavior indicates that the thermal diffusion time required for cooling to reach the indirect cooling region exceeded the 1 min contact duration. Under the 5 min condition, the temperature decreased by an additional approximately 1 °C after ice-pack removal, indicating that heat diffusion to the indirect cooling region remained incomplete. Because this post-removal decrease occurred without the cooling source and could be strongly affected by ambient conditions, the effective analysis interval was limited and was unsuitable for reliable feature extraction. In contrast, the additional decrease after ice-pack removal was no more than 0.5 °C under the 10 min condition, and both the cooling and recovery phases were stably captured, making this duration the most suitable for feature extraction. The 15 and 20 min conditions approached a quasi-steady state, with almost no additional decrease after ice-pack removal; however, the recovery interval was shortened, and the longer exposure of the ice pack to room air increased the likelihood of condensation. Because these longer durations offered little practical advantage over the 10 min condition, a 10 min ice-pack contact time was adopted as the final cooling-stimulus duration.

#### 2.3.3. Infrared Imaging and Data Acquisition

Each experiment continuously recorded an initial period of approximately 10 s, a 10 min cooling-stimulus period, and a recovery period of approximately 20 min, resulting in a total duration of approximately 30 min. The measurement procedure was as follows.

All specimens were thermally equilibrated under the relevant environmental conditions before infrared imaging; in this experiment, they were preconditioned for at least 12 h. During imaging, the relative positions of the camera and specimen were fixed, and the direct cooling region, indirect cooling region, and crumpled aluminum-foil area were included in the same frame. The direct cooling region covered more than half of the visible specimen area, and one specimen was imaged at a time. For chamber-preconditioned specimens, imaging began within 1 min after removal from the chamber to minimize temperature changes caused by exposure to the external environment.

After imaging began, the initial state was recorded for approximately 10 s, after which the ice pack was placed in contact with the direct cooling region for 10 min. No external compressive force was applied. Contact between the ice pack and the specimen surface was maintained solely by its self-weight. When manual stabilization was required to prevent slipping on the curved pipe surface, the ice pack was held without intentional compression outside the camera’s field of view. The specimen testing order was randomized to reduce systematic order effects associated with repeated ice-pack use. The circumferential contact region did not extend beyond approximately half of the pipe circumference, whereas the axial contact extent was constrained by the fixed length of the ice pack. The ice pack was removed after 10 min, and the specimen’s temperature recovery was then recorded continuously for approximately 20 min. After the 30 min recording was completed, the thermal data were transferred to a computer for post-processing.

The acquired thermographic data were analyzed using FLIR Thermal Studio (version 2.0.73; Teledyne FLIR, Wilsonville, OR, USA). ROIs were defined for the direct cooling region, indirect cooling region, and aluminum foil, and the required analysis parameters were specified. Time-series mean surface temperatures were then extracted from the direct and indirect cooling ROIs to analyze their thermal responses during cooling and recovery.

### 2.4. Thermal-Response Indicator Extraction and Statistical Analysis

#### 2.4.1. Estimation of the Recovery Time Constant *τ* in the Direct Cooling Region

**Figure 5:**
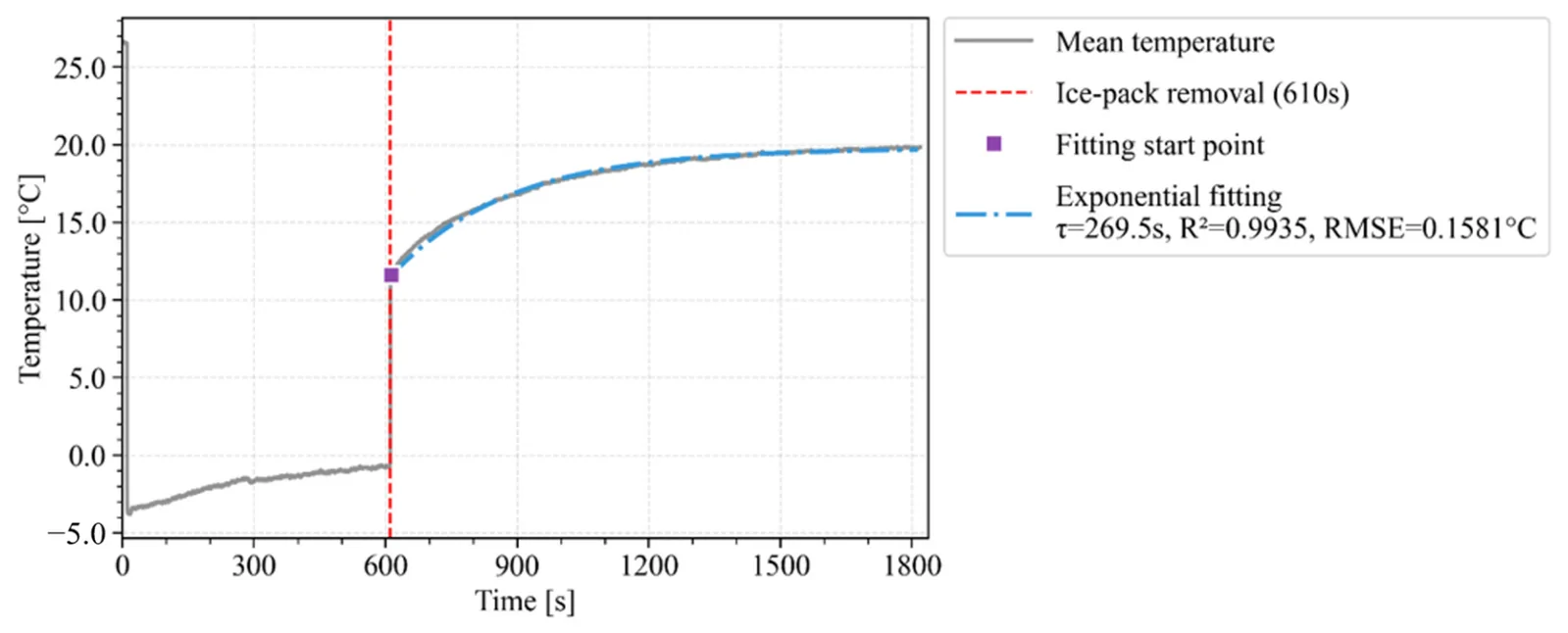
An example of an exponential model fitted to the recovery phase of the direct cooling region.

In the direct cooling region, where the ice pack contacted the steel pipe, the post-stimulus temperature–recovery curve was tracked to determine whether differences in recovery rate could discriminate non-corroded and corroded specimens. After ice-pack removal, this region exhibited a transient response as it returned toward ambient temperature through heat exchange with the surrounding air and the interior of the specimen. Because this behavior resembles a first-order lag response, it can be approximated using an exponential model. The time constant *τ*, defined as the time required to reach approximately 63% of the final temperature change, was therefore introduced as a single indicator of recovery rate. Because the NC specimens had relatively uniform metallic cross-sections and surface conditions, they were expected to exhibit more consistent heat-transfer behavior. In contrast, the faster recovery of the corroded specimens may have resulted from several potentially interacting factors, including changes in surface condition and roughness, alteration of the zinc coating, formation or delamination of corrosion products, interfacial voids, local differences in thermal contact, and possible metal loss. These factors may affect effective heat capacity, thermal conductivity, interfacial thermal resistance, and heat exchange with the surroundings. The exponential model in Equation (1) was fitted to the temperature recovery phase in the direct cooling region, and *τ* was used as the thermal-response indicator.

(1) $$T\left(t\right)=T_{\infty }-\left(T_{\infty }-T_{0}\right)e^{-t/\tau }$$

where $T_{\infty }$ is the temperature at the end of the recovery phase and $T_{0}$ is the temperature at the beginning of the recovery phase. $T_{\infty }$ was defined as the median temperature over the final 120 s of the recording to provide robustness against noise in the raw data. $T_{0}$ was determined automatically as the point at which a 0.2% slope change occurred after a temperature increase greater than the +2 °C threshold was detected following ice-pack removal. The time constant *τ* was the only free parameter and was estimated by nonlinear least squares using the scipy.optimize.curve_fit function. An example of the exponential-model fit is shown in Figure 5.

#### 2.4.2. Calculation of the Linearity Indices (*R*^2^ and RMSE) for the Cooling Phase in the Indirect Cooling Region

The indirect cooling region was adjacent to, but not in direct contact with, the ice pack. The analysis examined whether the linearity of its cooling phase could discriminate non-corroded and corroded specimens. Cooling in this region was transferred axially along the pipe interior and surface; consequently, the uniformity of heat transfer within the material was reflected in the shape of the cooling curve. A highly linear response was expected in the NC group because of its uniform heat-transfer path, whereas oxide layers, voids, and other corrosion-related features were expected to disturb heat transfer and reduce linearity in the corroded group.

**Figure 6:**
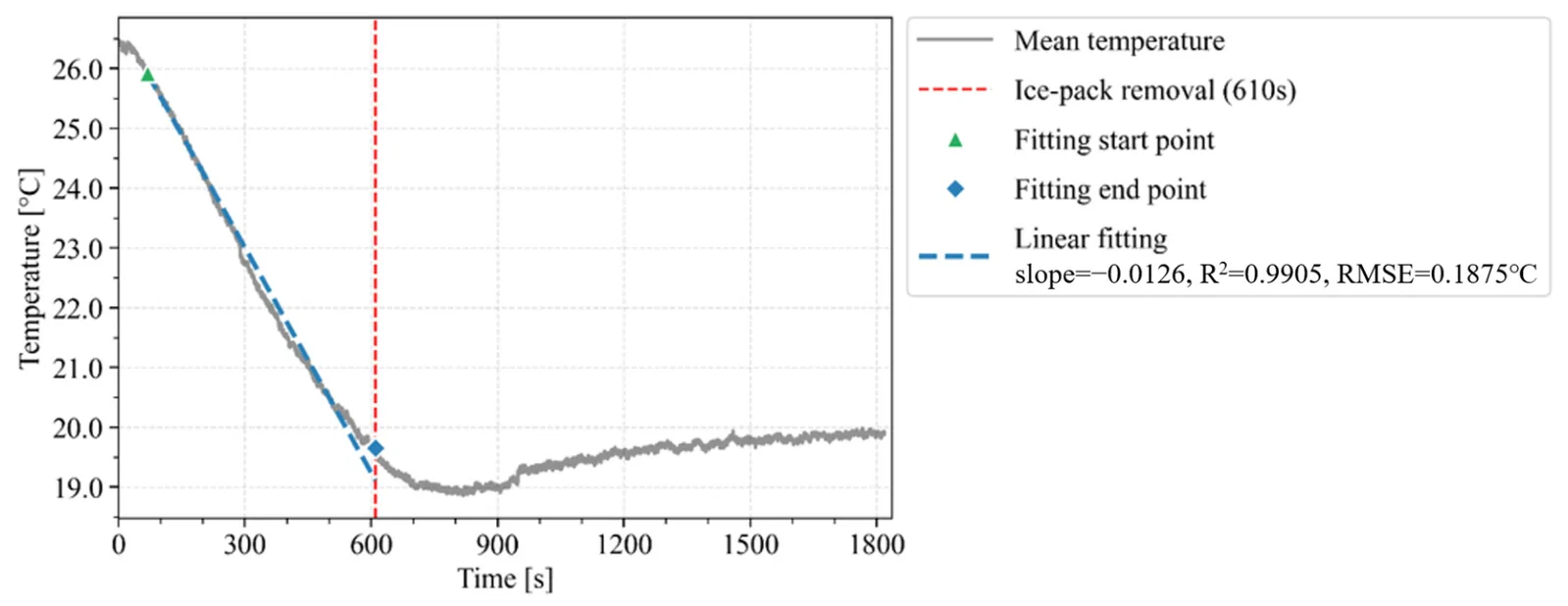
An example of a linear model fitted to the cooling phase of the indirect cooling region.

The linear model in Equation (2) was fitted from the start of ice-pack contact to the removal point, and *R*^2^ and RMSE were calculated. Here, $T_{0}$ denotes the temperature at the beginning of the fitted cooling interval, which was automatically defined as the point 0.5 °C below the peak after ice-pack contact to exclude the transient initial temperature increase and retain a stable cooling interval; κ denotes the fitted cooling slope. The endpoint was defined as the point at which the magnitude of the slope decreased to 30% or less of the maximum downward slope, thereby excluding the flattened portion near the minimum temperature. *R*^2^ is a dimensionless measure of linear goodness of fit, whereas RMSE quantifies the magnitude of the difference between the observations and fitted values. An example of the linear-model fit is shown in Figure 6. To evaluate the dependence of *R*^2^ on the data-dependent interval definition, a sensitivity analysis was performed using nine combinations of the start-temperature-drop threshold (0.3, 0.5, and 0.7 °C) and endpoint slope ratio (20%, 30%, and 40%). The combination of 0.5 °C and 30% corresponded to the baseline analysis.

(2) $$T\left(t\right)=T_{0}+\kappa \cdot t$$

During recovery in the indirect cooling region, convective heat exchange with the surrounding air becomes the primary heat-input pathway rather than axial conduction. The direct cooling region exhibited a distinct nonlinear exponential recovery response because of the large temperature difference after stimulus removal. In contrast, both cooling and recovery were gradual in the indirect cooling region, and the final temperature approached the ambient measurement temperature. The resulting temperature change during recovery was therefore limited, making the indirect-region recovery phase unsuitable for stable analysis.

#### 2.4.3. Statistical Analysis

The primary analytical comparison was between the NC group and the combined corroded group (SC + FC). The combined group was used as an operational binary screening category because the objective was to distinguish specimens without corrosion from specimens with a corrosion history, irrespective of the origin or duration of corrosion exposure. Comparisons of NC vs. SC, NC vs. FC, and SC vs. FC were treated as secondary exploratory comparisons. Each comparison was performed separately under each environmental condition, such that each specimen contributed only one observation to an individual test. Measurements obtained from the same specimen under different environmental conditions were not pooled as independent observations.

Differences in *τ* and RMSE were evaluated using Welch’s *t*-tests, and Cohen’s *d* was calculated to describe the standardized magnitude and direction of the observed differences. Because the *R*^2^ values were bounded between 0 and 1 and were concentrated near the upper boundary, their distributions could not be assumed to satisfy the conditions required for parametric analysis. Accordingly, comparisons of *R*^2^ were performed using exact two-sided Mann–Whitney U tests. Rank-biserial correlation was calculated as the corresponding effect-size measure. *R*^2^ values were summarized using the median and range (minimum–maximum).

Four group comparisons were performed for each of the four environmental conditions and three thermal-response indicators, resulting in a family of 48 statistical tests. The Holm procedure was applied across all 48 tests to control the family-wise Type I error rate. The family comprised Welch’s *p*-values for *τ* and RMSE and exact Mann–Whitney U *p*-values for *R*^2^. Statistical significance after applying the Holm procedure was defined as a Holm-adjusted *p*-value below 0.05. Given the small group sizes, all effect-size estimates were regarded as descriptive and were not interpreted as confirmatory evidence in isolation.

## 3. Results and Discussion

### 3.1. Qualitative Evaluation of Mean Surface-Temperature Trajectories

**Figure 7:**
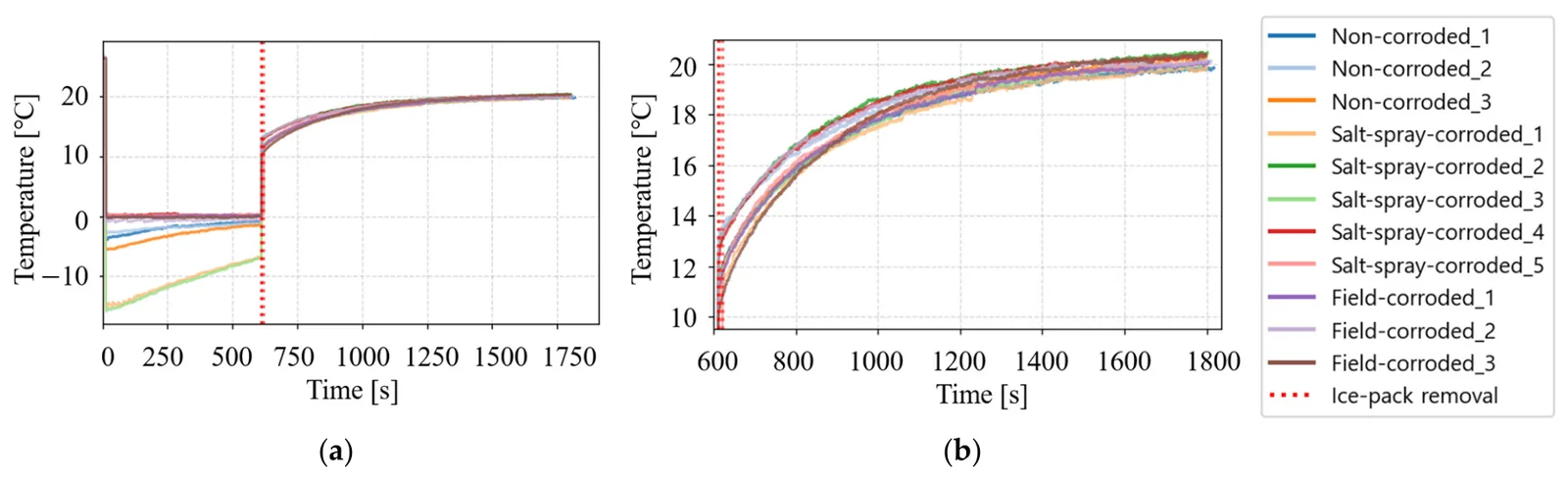
Mean surface-temperature trajectories for all specimens under the 27 °C condition: (**a**) In the entire direct cooling region; (**b**) Enlargement of the section after ice pack removal in the direct cooling region.

**Figure 8:**
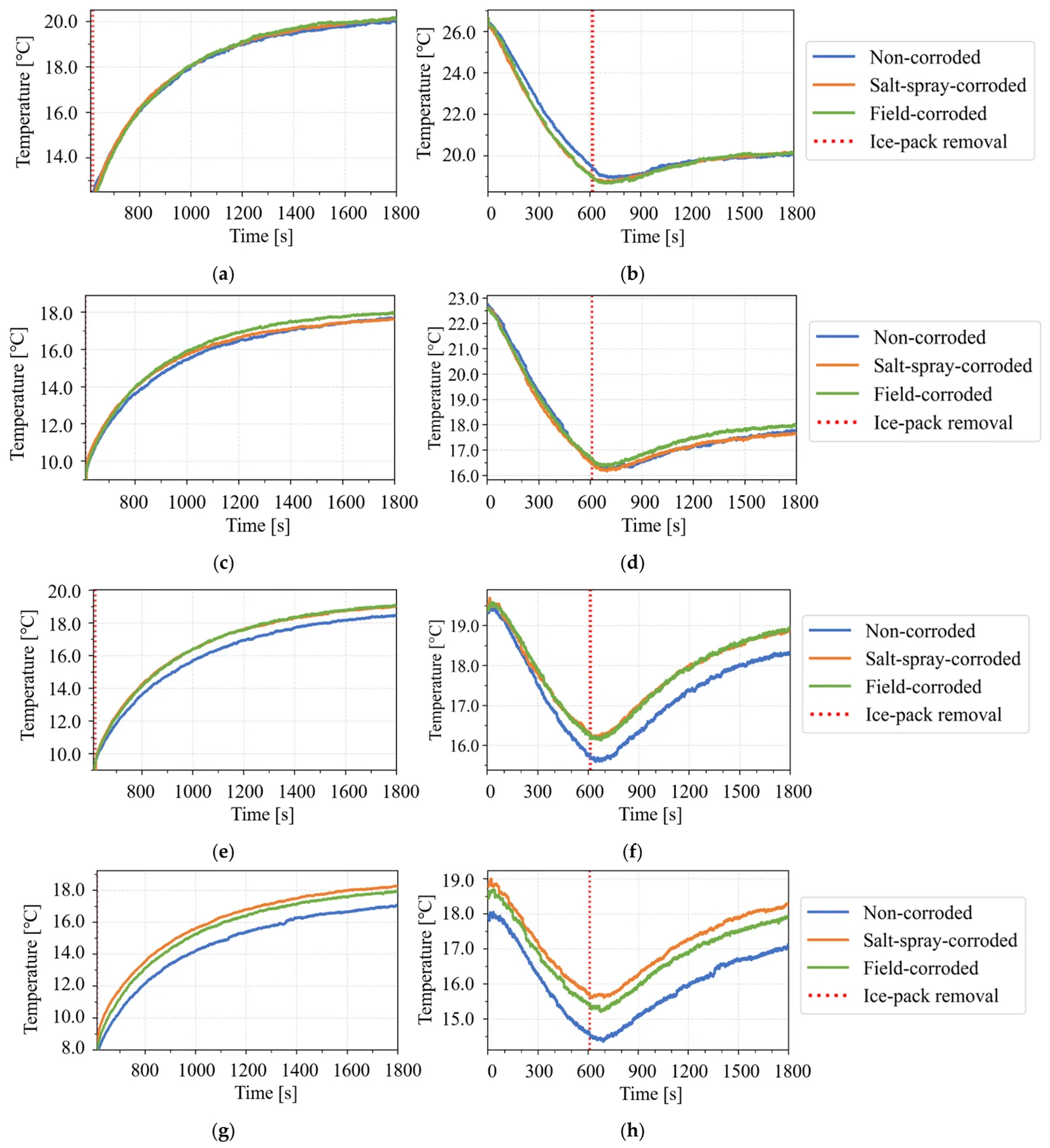
Mean surface-temperature trajectories of each group under each condition: (**a**) 27 °C in the direct cooling region; (**b**) 27 °C in the indirect cooling region; (**c**) 23 °C in the direct cooling region; (**d**) 23 °C in the indirect cooling region; (**e**) 19 °C in the direct cooling region; (**f**) 19 °C in the indirect cooling region; (**g**) RoomTemp 19 °C in the direct cooling region; (**h**) RoomTemp 19 °C in the indirect cooling region.

Time-series mean surface-temperature data for the direct cooling regions of all specimens measured under the 27 °C condition are shown in Figure 7. During the cooling phase, the ROI in the direct cooling region was obscured by the ice pack; consequently, the recorded temperature represented the ice-pack surface rather than the specimen and was excluded from the analysis. Temperature differences observed during this interval may reflect not only differences in ice-pack temperature caused by varying exposure to room air, but also the thermographic parameters set for the specimen and the moisture condition of the ice-pack surface. Plotting all specimen data over the full recording interval, as in Figure 7a, makes the overall thermal-response pattern difficult to discern. Figure 8 therefore presents the mean trajectories for each group, with the interval after ice-pack removal enlarged for the direct cooling region in the same manner as Figure 7b. Differences among environmental conditions represent differences in the initial temperatures of specimens equilibrated under each condition. In the direct cooling region, the post-removal recovery behavior was clearly observed, and recovery rates differed among groups. In the indirect cooling region, the temperature decrease during cooling was generally greater at higher initial specimen temperatures. Cooling proceeded more slowly as the initial specimen temperature approached room temperature. Although the initial temperature differed by 8 °C between the 27 °C and 19 °C conditions, the corresponding difference in minimum temperature was only approximately 5 °C, indicating that the minimum temperature did not follow the initial temperature in a one-to-one manner. During recovery, the difference between the minimum temperature and the final ambient temperature was approximately 3 °C under all environmental conditions, limiting the extraction of features with sufficient discriminatory power.

### 3.2. Recovery Time Constant τ in the Direct Cooling Region

When the exponential model was fitted to the recovery phase in the direct cooling region, the time constant *τ* consistently separated the NC and corroded groups across all environmental conditions. Shortening the exponential-model fitting interval could reduce the total measurement time. Accordingly, *τ* was calculated using four post-removal fitting windows of 5, 10, 15, and 20 min, where 20 min represented the entire recovery phase. Although the effect-size trends were similar among the four windows, Welch’s *p*-values and Cohen’s *d* values were most stable when *τ* was calculated from the full 20 min interval. This result supports the appropriateness of the 30 min recording constraint and the selected cooling and recovery durations. All subsequent *τ* values were therefore obtained by fitting the exponential model to the complete recovery phase.

**Figure 9:**
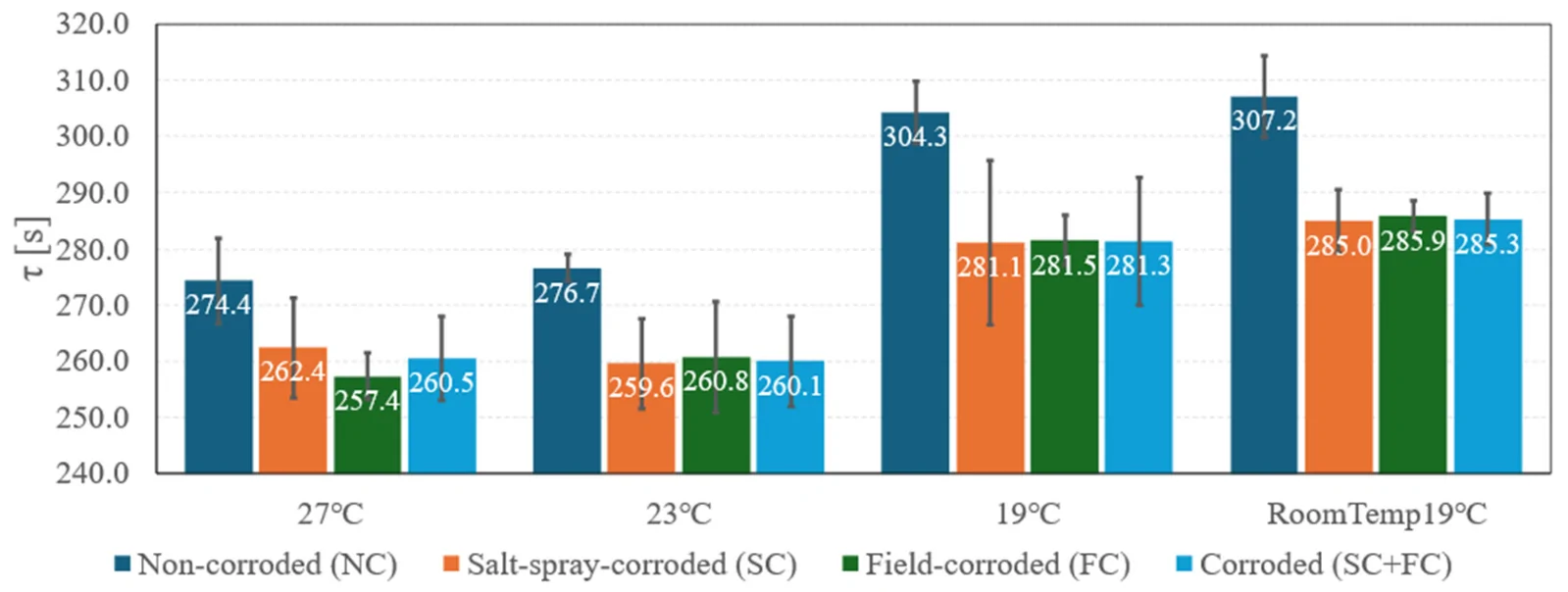
Comparison of mean thermal recovery time constants (*τ*) (±SD) among specimen groups.

The group-wise means of *τ* under each environmental condition are summarized in Table 6 and shown in Figure 9.

Under all four conditions, *τ* was largest in the NC group and smaller in the combined corroded group (SC + FC), indicating that corroded specimens recovered more rapidly than non-corroded specimens. The recovery time constant can be interpreted using a simplified lumped-capacitance approximation. If the responding pipe region is represented by an effective thermal capacitance *C_eff_* and an effective conductance *G_eff_* to its surroundings, the recovery response satisfies Equation (3), giving Equation (4). For a thin and approximately uniform pipe wall dominated by surface heat exchange, this relationship scales approximately as Equation (5), where *ρ*, *c*, *t*, and *h_eff_* denote density, specific heat, wall thickness, and effective surface heat-transfer coefficient, respectively. Wall-thickness loss could therefore reduce thermal mass and shorten *τ*.

(3) $$C_{eff}\frac{dT}{dt}={-G}_{eff}\left(T-T_{\infty }\right)$$

(4) $$\tau =\frac{C_{eff}}{G_{eff}}$$

(5) $$\tau \approx \frac{\rho ct}{h_{eff}}$$

However, corrosion-product layers may add thermal resistance and heat capacity, whereas changes in emissivity, roughness, and surface condition may alter radiative and convective heat exchange. Because these effects may act in opposing directions and were not measured independently, the lower *τ* observed in the corroded group cannot be attributed uniquely to wall-thickness loss. In addition, local cooling and axial conduction may violate the assumption of spatially uniform specimen temperature.

Under the 27 °C condition, mean *τ* was higher in the NC group than in the combined corroded group (unadjusted *p* = 0.060; *d* = 1.83). However, this difference did not reach the conventional significance level before correction and did not remain significant after applying the Holm procedure. Because the effect-size estimate was obtained from a small sample, the result was interpreted as a descriptive directional trend rather than statistical evidence of discrimination. At a higher initial specimen temperature, the specimen remained relatively warm after a fixed cooling duration; the smaller temperature difference between the start of recovery and room temperature consequently shortened the effective analysis interval and may have reduced statistical significance. Under the 23 °C, 19 °C, and RoomTemp 19 °C conditions, the *p* values were 0.001, 0.002, and 0.023, respectively, and the corresponding *d* values were 2.31, 2.23, and 4.17. Thus, mean *τ* was consistently higher in the NC group than in the combined corroded group across all four environmental conditions. Recovery was faster under the 27 °C and 23 °C conditions than under the 19 °C and RoomTemp 19 °C conditions, with a difference of approximately 30 s for the NC group. By contrast, the difference between the 27 °C and 23 °C conditions was only approximately 4 s, confirming that recovery rate was not directly proportional to initial specimen temperature.

### 3.3. Linearity Indices (R^2^ and RMSE) for the Cooling Phase in the Indirect Cooling Region

**Figure 10:**
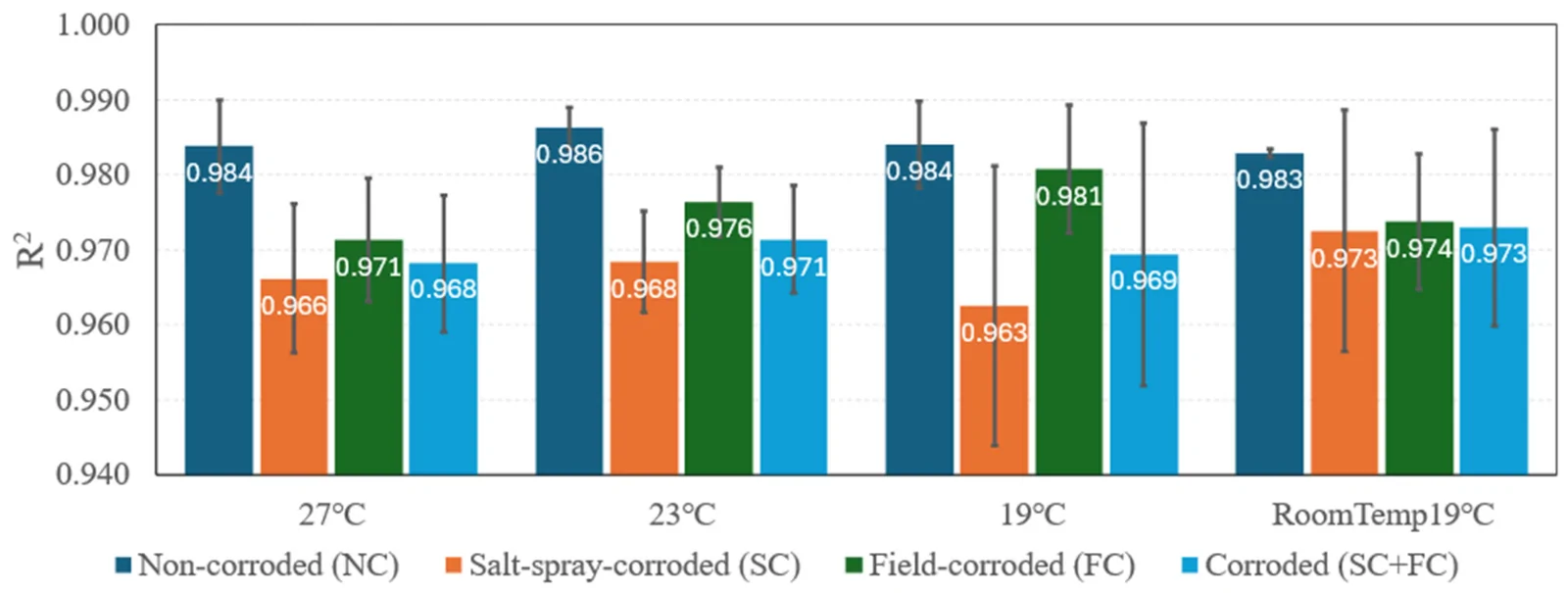
Comparison of mean *R*^2^ values (±SD) for the cooling phase among specimen groups.

**Figure 11:**
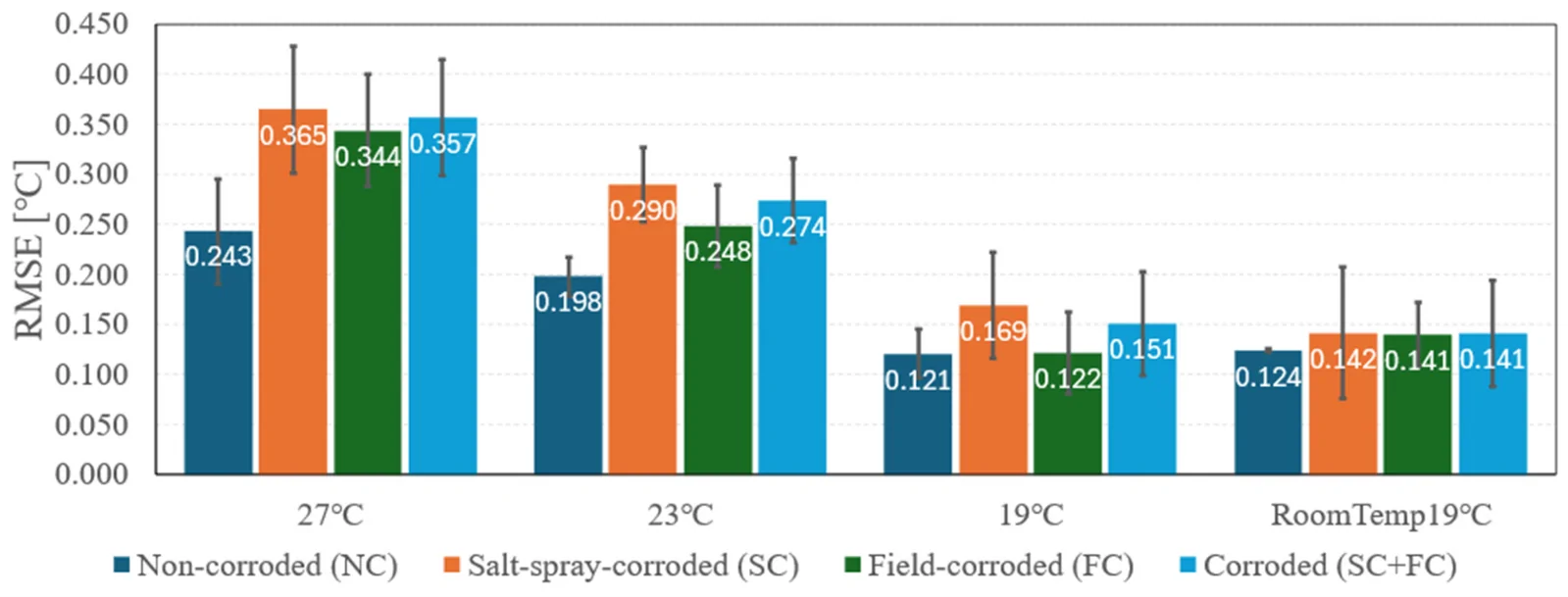
Comparison of mean RMSE values (±SD) for the cooling phase among specimen groups.

In the indirect cooling region, the cooling-curve linearity indices showed condition-dependent group differences. The group-wise results for *R*^2^ and RMSE under each environmental condition are summarized in Table 6 and shown in Figure 10 and Figure 11. The median *R*^2^ was higher in the NC group than in the combined corroded group under all four environmental conditions. RMSE was generally lower in the NC group, with the largest mean difference observed under the 27 °C condition.

For the primary comparison between the NC and combined corroded groups, exact two-sided Mann–Whitney U tests showed directional differences in *R*^2^ under the 27 °C and 23 °C conditions (unadjusted *p* = 0.012 for both conditions; *r*_rb_ = 1.000). An *r*_rb_ value of 1.000 indicates that all *R*^2^ values in the NC group were higher than those in the combined corroded group in the present samples. Under the 19 °C and RoomTemp 19 °C conditions, the corresponding unadjusted *p*-values were 0.194 and 0.133, and the *r*_rb_ values were 0.583 and 0.667, respectively. For RMSE, the unadjusted Welch’s *p*-values for the comparison between the NC and combined corroded groups were 0.036, 0.003, 0.227, and 0.392 under the 27 °C, 23 °C, 19 °C, and RoomTemp 19 °C conditions, respectively. Although some *R*^2^ and RMSE comparisons had unadjusted *p*-values below 0.05, none remained statistically significant after applying the Holm procedure. These results were therefore interpreted as exploratory condition-dependent differences.

Under the 27 °C and 23 °C conditions, the larger temperature decrease allowed differences in curve linearity to be reflected in both *R*^2^ and RMSE. The observed condition-specific trends suggest that the magnitude of temperature decrease may influence the linearity indices. Thus, the intensity of the cooling stimulus itself may determine discrimination performance and should be considered when designing an indirect cooling measurement protocol. Under the specific specimen, ice-pack, and environmental conditions evaluated in this experiment, the two higher-temperature conditions that produced nominally significant group differences were associated with a room-to-minimum temperature difference of approximately 6 °C or greater. This value represents an empirical range observed in the present dataset rather than a universal critical threshold.

In the indirect cooling region, the temperature response was governed primarily by axial heat transfer from the cooled region. A relatively uniform wall geometry and effective thermal diffusivity would be expected to produce a smooth cooling trajectory. Spatial variations in wall thickness, corrosion-product layers, interfacial thermal resistance, and surface boundary conditions could make the effective heat-transfer pathway heterogeneous and time-dependent, thereby increasing deviations from a simple linear fit. This provides a possible physical explanation for the generally lower *R*^2^ and higher RMSE observed in the corroded specimens. However, *R*^2^ and RMSE remain empirical descriptors of the selected fitting interval and cannot be assigned to a specific corrosion mechanism without independent measurements.

To evaluate the influence of the data-dependent interval definition, sensitivity analysis was performed using nine combinations of the start-temperature-drop threshold (0.3, 0.5, and 0.7 °C) and endpoint slope ratio (20%, 30%, and 40%). Across all nine combinations, the median *R*^2^ was higher in the NC group than in the combined corroded group under all four environmental conditions. For the primary comparison, the exact Mann–Whitney U *p*-values ranged from 0.012 to 0.085 under the 23 °C condition, from 0.012 to 0.048 under the 27 °C condition, from 0.194 to 0.497 under the 19 °C condition, and from 0.133 to 0.921 under the RoomTemp 19 °C condition. None of the *R*^2^ comparisons remained statistically significant after applying the Holm procedure in any of the nine threshold combinations. Thus, the direction of the observed *R*^2^ difference was consistent across the tested interval definitions, whereas the strength of the statistical evidence depended on the interval-selection criteria.

### 3.4. Statistical Comparison of Thermal-Response Indicators

The unadjusted statistical results for all 48 comparisons are presented in Table 6. Welch’s *p*-values and Cohen’s *d* estimates are reported for *τ* and RMSE, whereas exact two-sided Mann–Whitney U *p*-values and rank-biserial correlations are reported for *R*^2^. The recovery time constant *τ* was consistently higher in the NC group than in the combined corroded group across all four environmental conditions. The *R*^2^ and RMSE results showed condition-dependent directional differences, particularly under the 27 °C and 23 °C conditions. In contrast, none of the thermal-response indicators consistently distinguished the SC and FC groups at the present study scale.

After applying the Holm procedure across all 48 statistical tests, only the difference in *τ* between the NC and combined corroded groups under the 23 °C condition remained statistically significant (unadjusted Welch’s *p* = 0.000558; Holm-adjusted *p* = 0.0268). None of the *R*^2^ or RMSE comparisons retained statistical significance after applying the Holm procedure. In particular, the *R*^2^ comparisons between the NC and combined corroded groups under the 23 °C and 27 °C conditions both had unadjusted exact Mann–Whitney U *p*-values of 0.0121 and Holm-adjusted *p*-values of 0.5091. The remaining comparisons were therefore interpreted as exploratory.

Mean *τ* was consistently higher in the NC group than in the combined corroded group across all four environmental conditions. However, because the same specimens were evaluated under each condition and statistical significance after applying the Holm procedure was retained only under the 23 °C condition, this cross-condition consistency was interpreted as a descriptive directional pattern rather than as independent replication or evidence of statistically supported discrimination under all four conditions. Similarly, the directional differences in *R*^2^ and RMSE that did not remain significant after multiplicity adjustment were regarded as exploratory.

The principal difference between the 19 °C and RoomTemp 19 °C conditions was the magnitude of temperature variation. In the former, the initial specimen temperature was controlled within approximately ±0.1 °C, whereas in the latter the specimens were exposed to an indoor temperature variation of approximately 1 °C. The indirect-region linearity indices exhibited similar descriptive patterns under the 19 °C and RoomTemp 19 °C conditions. Although none of the corresponding group differences remained statistically significant after applying the Holm procedure, the median *R*^2^ was higher, and the mean RMSE was lower in the NC group than in the combined corroded group under both conditions. Mean *τ* was also higher in the NC group under both conditions. These similarities suggest that the modest initial-temperature variation evaluated in this study had a limited influence on the observed thermal-response patterns.

The limited discrimination between the SC and FC groups can be interpreted in relation to the composition of the two groups. The SC group contained stepwise exposure durations of 24–120 h and therefore did not represent a uniform degree of corrosion. Specimens with short exposure durations may have produced thermal responses closer to those of the NC group, whereas specimens with longer exposures may have approached the responses of the FC group. This heterogeneity likely increased within-group variance and reduced statistical power in the NC-vs.-SC and SC-vs.-FC comparisons. A further limitation of the FC group was that the actual degree of corrosion in each specimen was unknown. Under small-sample conditions, such specimen-to-specimen variation can strongly affect the results and make between-group discrimination difficult. Therefore, the present findings support only corrosion/non-corrosion screening and should not be interpreted as quantitative estimates of corrosion severity or remaining wall thickness.

## 4. Conclusions

This study designed a cold infrared thermography method for corrosion screening of greenhouse structural steel pipes and identified thermal-response indicators for different cooling-affected regions.

### 4.1. Design of the Cold Infrared Thermography Method

A principal operational advantage of the proposed method is that, although the cooling source directly contacts the specimen, the temperature measurement itself remains area-based and non-contact. Multiple ROIs can be observed simultaneously after a single stimulus, providing high information density. Thermographic data also facilitate temporal comparison, recording, and tracking at the same location and are therefore suitable for developing a maintenance-history database. The significance of this study lies in proposing a basic framework for corrosion screening of greenhouse structural steel pipes using a simple cooling source and area-based, non-contact measurement. The principal elements of the measurement method are as follows.

- A high-emissivity reference surface should be used to reduce emissivity-related error on metallic surfaces. In this study, high-emissivity insulation tape was applied to each specimen ROI to equalize emissivity.
- A specimen fixture should be selected to minimize temperature disturbance caused by external contact.
- The cooling-source material and surface treatment should be selected to minimize condensation, and appropriate handling and storage procedures should be established.
- The total acquisition time and cooling-stimulus duration should be selected by considering both the need to capture stable cooling and recovery phases and the practical constraints of field measurement.

### 4.2. Thermal-Response Indicators for Corrosion Screening

The ice-pack measurement method was applied to greenhouse structural steel pipes, and thermal-response indicators were evaluated separately in the direct and indirect cooling regions. The time constant *τ* was introduced as an indicator of recovery rate in the direct cooling region, whereas *R*^2^ and RMSE were introduced as linearity indices describing the cooling-curve shape in the indirect cooling region. Four initial-temperature conditions (27 °C, 23 °C, 19 °C, and RoomTemp 19 °C) were used to evaluate the influence of initial temperature on corrosion discrimination. The principal conclusions are as follows.

- The recovery-phase time constant *τ* in the direct cooling region was larger in the NC group than in the corroded group, indicating that corroded specimens recovered more rapidly. After applying the Holm procedure across all 48 comparisons, the group difference remained statistically significant only under the 23 °C condition.
- The indirect-region linearity indices showed condition-dependent directional differences between the NC and combined corroded groups. The direction of the *R*^2^ difference was consistent across the tested interval-selection criteria, but its statistical strength depended on the interval definition. None of the *R^2^* or RMSE comparisons remained statistically significant after applying the Holm procedure; these indices were therefore regarded as exploratory and procedure-dependent indicators requiring further validation.
- Discrimination among the detailed specimen groups, other than the comparison between the NC and combined corroded groups, was limited at the present sample size.

### 4.3. Limitations and Future Work

This study included only three NC, five SC, and three FC specimens and should therefore be regarded as a proof-of-concept evaluation. With these small group sizes, the Welch’s *p*-values and Cohen’s *d* estimates for *τ* and RMSE, as well as the exact Mann–Whitney U *p*-values and rank-biserial correlations for *R*^2^, are subject to substantial sampling uncertainty. The Holm procedure was applied across all 48 statistical tests, including the primary comparison between the NC and combined corroded groups and the secondary pairwise comparisons. After applying the Holm procedure, only *τ* under the 23 °C condition remained statistically significant for the primary comparison. The remaining differences were regarded as exploratory. Validation using a substantially larger and independently collected specimen set is therefore required. *R*^2^ was also partly dependent on the data-driven definition of the fitted interval. Although the direction of the difference between the NC and combined corroded groups was consistent across the tested threshold combinations, the exact Mann–Whitney U *p*-values varied according to the interval-selection criteria. *R*^2^ should therefore be regarded as a procedure-dependent exploratory indicator until its robustness is validated using an independently collected dataset and a prespecified interval-selection protocol.

The same specimens were evaluated under all four environmental conditions; therefore, the condition-specific results were correlated and should not be interpreted as independent replications. In addition, within-group variance in the SC group may have been increased because a different salt-spray exposure duration was assigned to each specimen. Future experiments should include replicate specimens at each exposure duration to characterize and control this source of variability. Corrosion severity should also be quantified using independent reference measurements, such as mass loss, wall-thickness loss, corroded surface area, or ultrasonic thickness, to investigate its quantitative relationship with the thermal-response indicators. Previous sensing-based research has demonstrated that quantitative corrosion assessment requires a physical model linking the sensor response to corrosion parameters and validation against independent measurements such as pit depth and mass loss [27].

The ice-pack contact area, contact pressure, and initial interface temperature were not quantitatively measured, and replicate experiments were not conducted to assess variability associated with the ice-pack contact conditions. Although randomization reduced systematic order effects, it may not have completely eliminated application-to-application variability. Future studies should evaluate measurement reproducibility using standardized ice-pack contact conditions and replicate measurements. Furthermore, the fixed imaging distance, common pipe diameter, apex-centered ROI placement, and high-emissivity tape reduced variations in viewing geometry and emissivity among specimens. However, the view-angle dependence of the apparent temperature on the cylindrical surface was not independently quantified and should be evaluated in future validation.

The current procedure requires 10 min of cooling, approximately 20 min of recovery, and application of high-emissivity tape to each ROI. It should therefore be regarded as a proof-of-concept screening prototype rather than a field-ready inspection system. Large-scale implementation will require shorter acquisition times, rapid and repeatable application of the emissivity reference surface, standardized cooling-source contact, and evaluation of inspection throughput. Furthermore, measurements in actual greenhouses may be affected by reflected radiation, background-temperature variations, wind, and surface contamination. Field validation is therefore required to calibrate the measurement conditions and refine the screening criteria.

## Figures and Tables

**Table 1:** Summary of specimen types, specifications, and characteristics.

Group	Name	Thickness (mm)	Length (mm)	Source/Fabrication	Exposure Conditions
NC ^1^	NC1	1.5 (nominal)	500	Cutting of new steel pipe	(Indoor storage after cutting)
	NC2				
	NC3				
SC ^2^	SC1	1.5 (nominal, before salt-spray exposure)	500	KS D 9502 salt spray test	24 h
	SC2				48 h
	SC3				72 h
	SC4				96 h
	SC5				120 h
FC ^3^	FC1	Unknown (not measured before field exposure)	570	Collected from discarded greenhouse steel pipes	Natural corrosion (over 10 years)
	FC2		710		
	FC3		640		

^1^ Non-corroded; ^2^ Salt-spray-corroded; ^3^ Field-corroded.

**Table 2:** Key specifications of the infrared thermal camera.

Manufacturer	Product Name	IR Resolution	Thermal Sensitivity	Accuracy	Field of View
Teledyne FLIR	T560	640 × 480 pixels	<40 mK	±2 °C (±3.6 °F) or ±2% of reading	24° × 18°

**Table 3:** Specifications of the insulation tape utilized for emissivity equalization.

Manufacturer	Product Name	Operating Temperature Range (°C)	Color	Width (mm)	Thickness (mm)
3M Company	Scotch Super 88	−18–105	Black	19	0.216

**Table 4:** Specifications of the constant temperature and humidity chamber utilized for specimen initial temperature setting.

Manufacturer	Product Name	Temperature (°C)			Relative Humidity (% RH)		
		Range	Accuracy	Resolution	Range	Accuracy	Resolution
DOOSUNG	SH-LB-3	2–48	±0.1	0.01	25–95	±1.0	0.10

**Table 5:** Nomenclature for each initial temperature setting of the specimen.

Description	Environmental Conditions
27 °C	27 °C, 50% RH
23 °C	23 °C, 50% RH
19 °C	19 °C, 50% RH
RoomTemp 19 °C	18–19 °C, 33–38% RH

**Table 6:** Group-wise comparisons of the recovery time constant (*τ*) and cooling-phase linearity indices (*R*^2^ and RMSE) under the four environmental conditions. The comparison between the non-corroded (NC) group and the combined corroded group comprising salt-spray-corroded (SC) and field-corroded (FC) specimens was the main comparison of interest; Welch’s *p*-values and Cohen’s *d* estimates are reported for *τ* and RMSE; exact Mann–Whitney U *p*-values and rank-biserial correlations (*r*_rb_) are reported for *R*^2^. All *p*-values shown in the table are unadjusted.

Conditions	Comparison Group	n	Mean ± SD of *τ* (s)	Unadjusted Welch’s *p*-Value and Cohen’s *d*	Median [Range] of *R*^2^	Unadjusted Exact Mann–Whitney U *p*-Value and Rank-Biserial Correlation (*r*_rb_)	Mean ± SD of RMSE (°C)	Unadjusted Welch’s *p*-Value and Cohen’s *d*
27 °C	NC ^1^ vs. (SC ^2^ + FC ^3^)	3 vs. 8	274.39 ± 7.58 vs. 260.55 ± 7.56	*p* = 0.060, *d* = 1.83	0.983 [0.978–0.990] vs. 0.971 [0.954–0.978]	*p* = 0.012, *r*_rb_ = 1.000	0.243 ± 0.053 vs. 0.357 ± 0.058	*p* = 0.036, *d* = −2.02
	NC vs. SC	3 vs. 5	274.39 ± 7.58 vs. 262.43 ± 8.93	*p* = 0.100, *d* = 1.41	0.983 [0.978–0.990] vs. 0.970 [0.954–0.977]	*p* = 0.036, *r*_rb_ = 1.000	0.243 ± 0.053 vs. 0.365 ± 0.063	*p* = 0.032, *d* = −2.03
	NC vs. FC	3 vs. 3	274.39 ± 7.58 vs. 257.41 ± 4.09	*p* = 0.040, *d* = 2.79	0.983 [0.978–0.990] vs. 0.974 [0.962–0.978]	*p* = 0.100, *r*_rb_ = 1.000	0.243 ± 0.053 vs. 0.344 ± 0.056	*p* = 0.086, *d* = −1.85
	SC vs. FC	5 vs. 3	262.43 ± 8.93 vs. 257.41 ± 4.09	*p* = 0.321, *d* = 0.66	0.970 [0.954–0.977] vs. 0.974 [0.962–0.978]	*p* = 0.393, *r*_rb_ = −0.467	0.365 ± 0.063 vs. 0.344 ± 0.056	*p* = 0.646, *d* = 0.35
23 °C	NC vs. (SC + FC)	3 vs. 8	276.66 ± 2.45 vs. 260.07 ± 8.06	*p* = 0.001, *d* = 2.31	0.985 [0.984–0.989] vs. 0.971 [0.959–0.981]	*p* = 0.012, *r*_rb_ = 1.000	0.198 ± 0.020 vs. 0.274 ± 0.042	*p* = 0.003, *d* = −2.01
	NC vs. SC	3 vs. 5	276.66 ± 2.45 vs. 259.61 ± 8.02	*p* = 0.007, *d* = 2.54	0.985 [0.984–0.989] vs. 0.969 [0.959–0.977]	*p* = 0.036, *r*_rb_ = 1.000	0.198 ± 0.020 vs. 0.290 ± 0.037	*p* = 0.004, *d* = −2.83
	NC vs. FC	3 vs. 3	276.66 ± 2.45 vs. 260.83 ± 9.85	*p* = 0.100, *d* = 2.21	0.985 [0.984–0.989] vs. 0.977 [0.972–0.981]	*p* = 0.100, *r*_rb_ = 1.000	0.198 ± 0.020 vs. 0.248 ± 0.041	*p* = 0.154, *d* = −1.57
	SC vs. FC	5 vs. 3	259.61 ± 8.02 vs. 260.83 ± 9.85	*p* = 0.097, *d* = −1.30	0.969 [0.959–0.977] vs. 0.977 [0.972–0.981]	*p* = 0.143, *r*_rb_ = −0.733	0.290 ± 0.037 vs. 0.248 ± 0.041	*p* = 0.220, *d* = 1.09
19 °C	NC vs. (SC + FC)	3 vs. 8	304.33 ± 5.49 vs. 281.25 ± 11.36	*p* = 0.002, *d* = 2.23	0.986 [0.978–0.988] vs. 0.976 [0.942–0.990]	*p* = 0.194, *r*_rb_ = 0.583	0.121 ± 0.024 vs. 0.151 ± 0.052	*p* = 0.227, *d* = −0.65
	NC vs. SC	3 vs. 5	304.33 ± 5.49 vs. 281.11 ± 14.69	*p* = 0.022, *d* = 1.87	0.986 [0.978–0.988] vs. 0.955 [0.942–0.983]	*p* = 0.143, *r*_rb_ = 0.733	0.121 ± 0.024 vs. 0.169 ± 0.053	*p* = 0.135, *d* = −1.05
	NC vs. FC	3 vs. 3	304.33 ± 5.49 vs. 281.49 ± 4.46	*p* = 0.006, *d* = 4.57	0.986 [0.978–0.988] vs. 0.976 [0.975–0.990]	*p* = 0.700, *r*_rb_ = 0.333	0.121 ± 0.024 vs. 0.122 ± 0.040	*p* = 0.984, *d* = −0.02
	SC vs. FC	5 vs. 3	281.11 ± 14.69 vs. 281.49 ± 4.46	*p* = 0.959, *d* = −0.03	0.955 [0.942–0.983] vs. 0.976 [0.975–0.990]	*p* = 0.393, *r*_rb_ = −0.467	0.169 ± 0.053 vs. 0.122 ± 0.040	*p* = 0.210, *d* = 0.96
RoomTemp 19 °C	NC vs. (SC + FC)	3 vs. 8	307.18 ± 7.29 vs. 285.30 ± 4.50	*p* = 0.023, *d* = 4.17	0.983 [0.982–0.983] vs. 0.977 [0.953–0.989]	*p* = 0.133, *r*_rb_ = 0.667	0.124 ± 0.002 vs. 0.141 ± 0.053	*p* = 0.392, *d* = −0.37
	NC vs. SC	3 vs. 5	307.18 ± 7.29 vs. 284.95 ± 5.59	*p* = 0.015, *d* = 3.58	0.983 [0.982–0.983] vs. 0.981 [0.953–0.989]	*p* = 0.250, *r*_rb_ = 0.600	0.124 ± 0.002 vs. 0.142 ± 0.066	*p* = 0.585, *d* = −0.33
	NC vs. FC	3 vs. 3	307.18 ± 7.29 vs. 285.88 ± 2.78	*p* = 0.025, *d* = 3.86	0.983 [0.982–0.983] vs. 0.973 [0.965–0.983]	*p* = 0.200, *r*_rb_ = 0.778	0.124 ± 0.002 vs. 0.141 ± 0.032	*p* = 0.472, *d* = −0.72
	SC vs. FC	5 vs. 3	284.95 ± 5.59 vs. 285.88 ± 2.78	*p* = 0.767, *d* = −0.19	0.981 [0.953–0.989] vs. 0.973 [0.965–0.983]	*p* = 1.000, *r*_rb_ = −0.067	0.142 ± 0.066 vs. 0.141 ± 0.032	*p* = 0.971, *d* = 0.023

^1^ Non-corroded; ^2^ Salt-spray-corroded; ^3^ Field-corroded.

## Data Availability

The data presented in this study are available on request from the corresponding author.

## Abbreviations

The following abbreviations are used in this manuscript:

RMSE	Root mean square error
NC	Non-corroded
SC	Salt-spray-corroded
FC	Field-corroded
IRT	Infrared thermography
ROI	Region of interest
RH	Relative humidity
CSQ	Compressed Sequence
DRR	Dynamic reference reconstruction
DTW	Dynamic time warping
PCM	Phase-change material
SD	Standard deviation

## References

[1] Maraveas C. (2020). Durability Issues and Corrosion of Structural Materials and Systems in Farm Environment. Appl. Sci..

[2] Wei H., Tang Y., Liu Y.-P., Chan S.-L. (2025). Research on durability and mechanical properties of high strength steel in corrosive environment: A review. Structures.

[3] Yun S.W., Choi M.K., Lee S.Y., Moon S.D., Yoon Y.C. (2015). Corrosion Rate of Structural Pipes for Greenhouse. J. Bio-Environ. Control.

[4] Morgan J., Rubaiyat A., Judd K., Thai D., Tagert J., Rohde G. (2025). Corrosion detection from IR thermal images in signed cumulative distribution transform domain. NDT E Int..

[5] Wicker M., Alduse B., Jung S. (2018). Detection of hidden corrosion in metal roofing shingles utilizing infrared thermography. J. Build. Eng..

[6] Nam S.W., Ryu H.R., Choi M.K., Shin H.H. (2020). Corrosion and Strength Changes of Agricultural Steel Pipes Elapsed 20 Years under the Greenhouse Environment. J. Bio-Environ. Control.

[7] Zou Z., Li W., Li Q., Jia S., Zhou Y. (2025). Machine Learning-Assisted Early-Corrosion Detection System for Pipeline Coatings. IEEE Sens. J..

[8] Egodawela S., Gostar A., Buddika H., Harischandra W., Dhammika A., Mahmoodian M. (2025). Metal loss defect detection and depth estimation using multi-spectral image analysis of cooling excited steel specimen with corrosion. Sci. Rep..

[9] Vasagar V., Hassan M.K., Abdullah A.M., Karre A.V., Chen B., Kim K., Al-Qahtani N., Cai T. (2024). Non-destructive techniques for corrosion detection: A review. Corros. Eng. Sci. Technol..

[10] Usamentiaga R., Venegas P., Guerediaga J., Vega L., Molleda J., Bulnes F.G. (2014). Infrared Thermography for Temperature Measurement and Non-Destructive Testing. Sensors.

[11] Doshvarpassand S., Wu C., Wang X. (2019). An overview of corrosion defect characterization using active infrared thermography. Infrared Phys. Technol..

[12] Elizalde J., Chiou Y. (2023). Improving the detectability and confirmation of hidden surface corrosion on metal sheets using active infrared thermography. J. Build. Eng..

[13] Mulaveesala R., Arora V., Rani A. (2019). Coded thermal wave imaging technique for infrared non-destructive testing and evaluation. Nondestruct. Test. Eval..

[14] Rezayiye R., Ibarra-Castanedo C., Maldague X. (2024). Methods for Corrosion Detection in Pipes Using Thermography: A Case Study on Synthetic Datasets. Algorithms.

[15] Doshvarpassand S., Wang X., Zhao X. (2022). Sub-surface metal loss defect detection using cold thermography and dynamic reference reconstruction (DRR). Struct. Health Monit..

[16] Doshvarpassand S., Wang X. (2022). Sub-Surface Defect Depth Approximation in Cold Infrared Thermography. Sensors.

[17] (2023). Coated Steel Pipes for Vinyl Housing.

[18] (2024). Neutral, Acetic Acid and Copper-Accelerated Acetic Acid Salt Spray.

[19] Teledyne FLIR Use Low-Cost Materials to Increase Target Emissivity. https://www.flir.com/discover/rd-science/use-low-cost-materials-to-increase-target-emissivity/.

[20] Avdelidis N.P., Moropoulou A. (2003). Emissivity considerations in building thermography. Energy Build..

[21] Cheng C., Chen D., Shao S., Na R., Cai H., Zhou H., Wu B. (2025). Revealing the Impact of Depth and Surface Property Variations on Infrared Detection of Delamination in Concrete Structures Under Natural Environmental Conditions. Buildings.

[22] Giasin K., Ayvar-Soberanis S. (2016). Evaluation of Workpiece Temperature During Drilling of GLARE Fiber Metal Laminates Using Infrared Techniques: Effect of Cutting Parameters, Fiber Orientation and Spray Mist Application. Materials.

[23] Mahmoodzadeh M., Gretka V., Lee I., Mukhopadhyaya P. (2022). Infrared thermography for quantitative thermal performance assessment of wood-framed building envelopes in Canada. Energy Build..

[24] Sommer K., Plez B., Cohen-Tanugi J., Dagoret-Campagne S., Moniez M., Neveu J., Betoule M., Bongard S., Feinstein F., Le Guillou L. (2024). StarDICE II: Calibration of an Uncooled Infrared Thermal Camera for Atmospheric Gray Extinction Characterization. Sensors.

[25] Teledyne FLIR Reference Documentation Thermography. https://support.flir.com/DSDownload/Assets/T810442-en-US_A4.pdf.

[26] (1976). Standard Atmospheres for Conditioning and/or Testing—Specifications.

[27] Xu L., Shi S., Huang Y., Yan F., Wang X., Wilson R., Zhang D. (2025). Quantification and assessment of steel pitted corrosion using optical frequency domain reflectometry (OFDR)-based distributed fiber optic sensors. Measurement.

